# Supplementary material for: Spike conformational and glycan heterogeneity associated with furin cleavage causes incomplete neutralization of SARS-CoV-2
Source: Nat Commun. 2025 Nov 19;16:10130. doi: 10.1038/s41467-025-65099-y (PMC12630640; doi:10.1038/s41467-025-65099-y)
Supplement: Supplementary file 1 — Supplementary Information [file 41467_2025_65099_MOESM1_ESM.pdf]

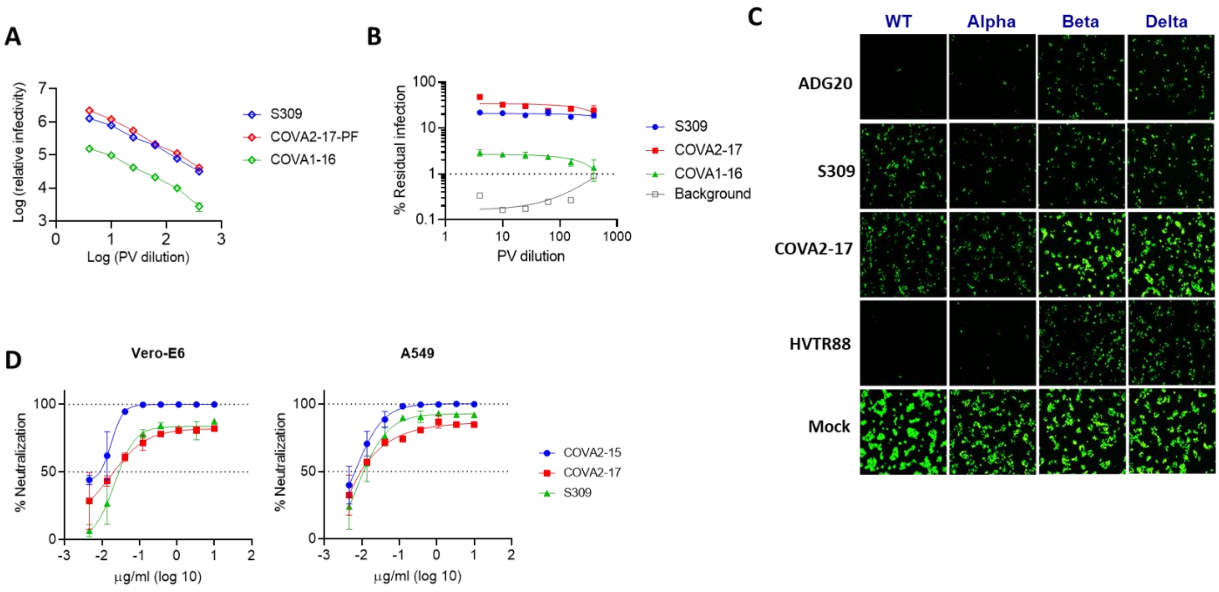

Figure S1: The PF in neutralization of SARS-CoV-2 variant pseudoviruses. A. Relative residual infectivity of WT in the presence of excess concentration of indicated nAbs (50 µg/ml) is plotted as a function of log10 of varying virus inoculum. These nAbs were chosen because of their atypical neutralization that saturates below 100%. B. Percent neutralization of WT at varying inoculums by indicated nAbs is plotted. C. The residual infection of various eGFP expressing pseudovirus in the presence of indicated nAbs. D. Dose-dependent neutralization of WT pseudovirus by the indicated antibodies measured using Vero-E6 and A549 cells.

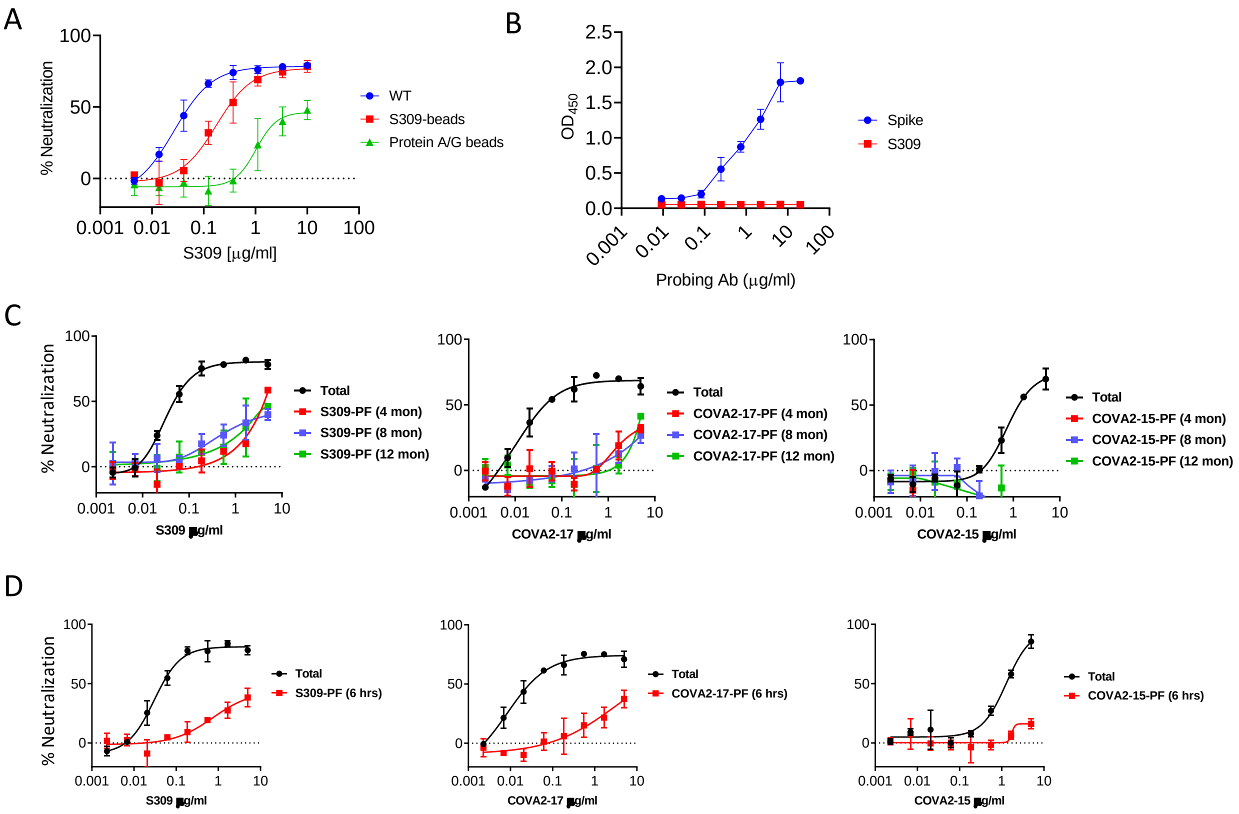

Figure S2: The virus depletion and assessment of depleted pseudovirus: A. Neutralization sensitivity of undepleted and depleted (PF) virus to S309. The depletion was either done by using covalently coupled S309 beads or soluble S309 followed by capture of neutralized virions by protein A/G beads (related to figure 2). B. Detection of the presence of residual S309 in the PF. The OD at 450nm is plotted from ELISA detecting viral spike probed with anti-spike mouse serum IgG and S309 was probed directly with anti-human-HRP conjugate. C. Neutralization sensitivity of undepleted and depleted WT after storage at -80°C for months. The sensitivity of various PFs to the depleting antibodies is shown. D. Neutralization sensitivity of undepleted WT and its PF after incubating the virus at 37°C for up to 6 hours.

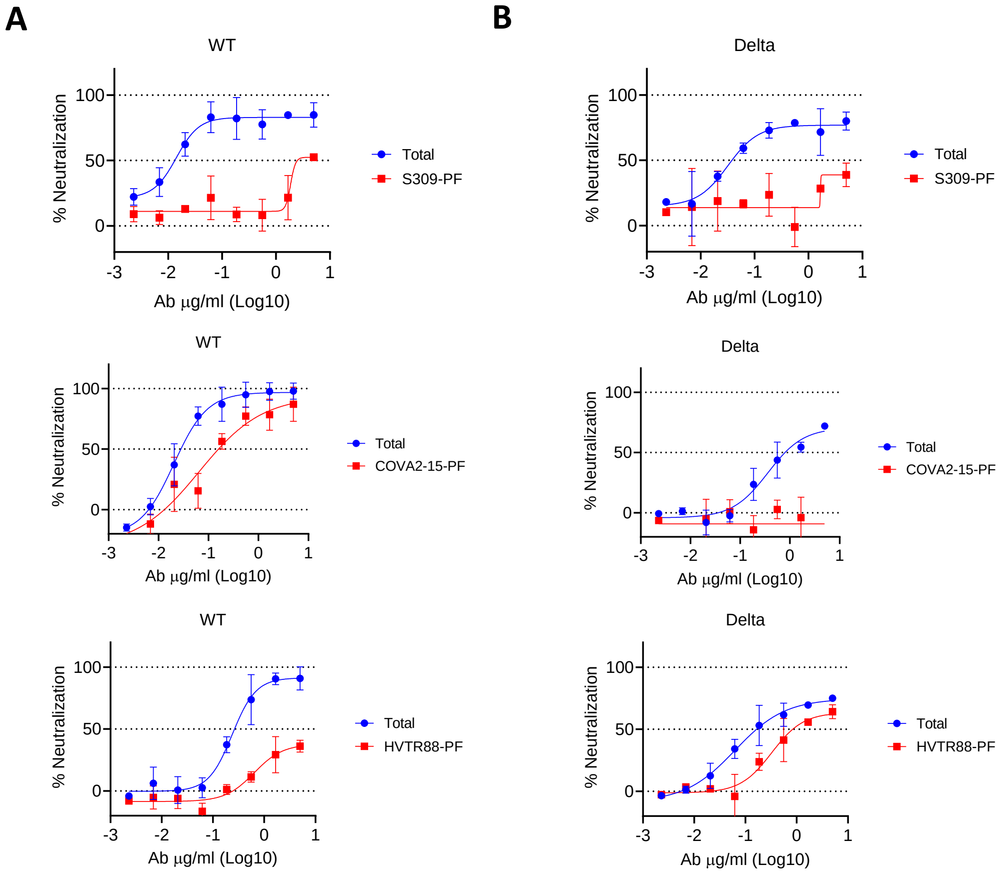

Figure S3: The depletion of Ab-sensitive authentic virus from the total population to isolate PF: A. The authentic WT virus was prepared in Vero-TMPRSS2 cells and virus sock of passage-2 was used for the depletion experiment. The depletion was done by S309, COVA2-15 or HVTR88 and was tested against the same antibodies after depletion. Shown are the neutralization patterns of the total population and respective PFs against the depleting antibodies. B. The same data as in A but for Delta virus. This virus was also prepared in the Vero-TMPRSS2 cells and passage-3 was used for the depletion.

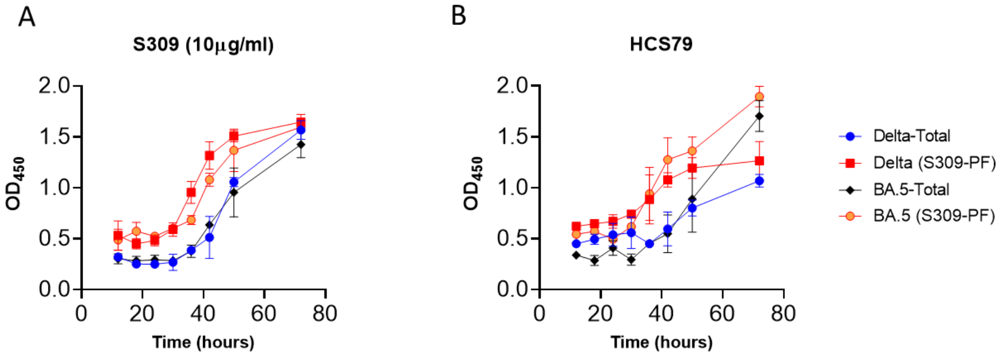

Figure S4: SARS-CoV-2 replication kinetics in the neutralizing conditions: A. Replication of authentic virus (Delta and BA.5) and their S309-PF in the presence of S309 at 10µg/ml S309 or a potent neutralizing serum HCS-79. The infection of Vero-TMPRSS2 cells was measured by fixing the cells and detecting intracellular viral nucleocapsid protein in ELISA.

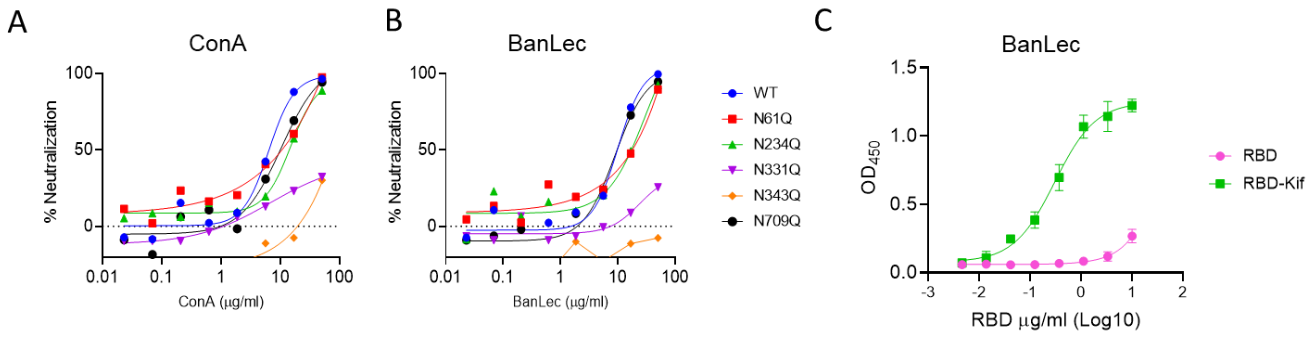

Figure S5: Neutralization sensitivity of pseudovirus to lectins and depletion of Spike ectodomain by ConA: A, B. The neutralization sensitivity of WT and its glycan knock-out mutants to ConA (A) and BanLec (B). C. Binding of RBD-Kif and RBD protein to BanLec in ELISA. The RBD-Kif was expressed in the expi293 cells in the presence of Kifunensine and RBD without Kifunensine; the former is supposed to contain mannosidic glycans at N331 and N343 residues.

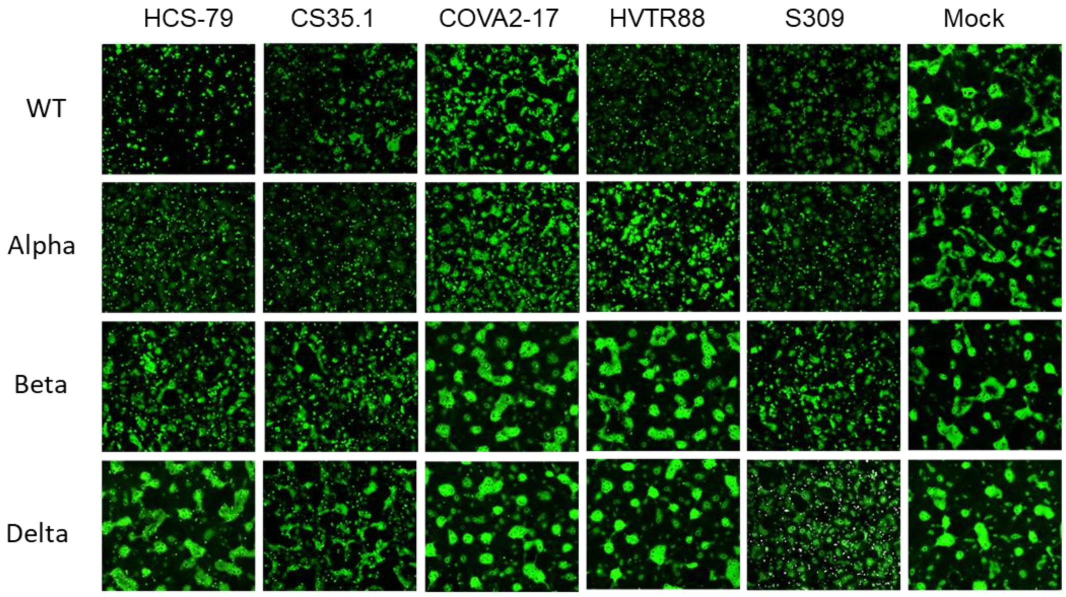

Figure S6: Spike fusogenicity in the presence of nAbs: The fusion of spike-expressing cells with Vero-TMPRSS2 cells is shown in the presence of excess concentration (100µg/ml) of monoclonal antibody or 1:10 dilution of human serum HCS-79 (first wave) or CS35.1 (third wave). The fusion was recorded after 1 hour incubation of spike-transfected cells with Vero cells. Mock represents the control in which neutralizing antibody was not added.

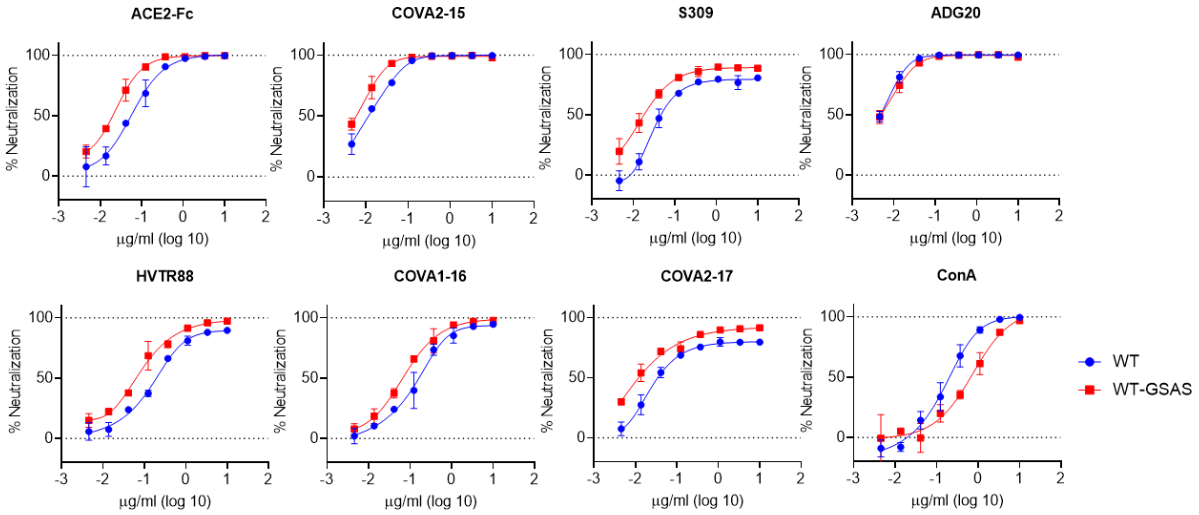

Figure S7: Neutralization sensitivity of WT and WT-GSAS pseudovirus: Shown are the neutralization curves of WT and mutant pseudovirus (furin cleavage site knock-out) for ACE2-Fc, monoclonal neutralizing antibodies, and lectin ConA. The percent neutralization is plotted on Y-axis against concentration of neutralizer on x-axis.

Table S1: The infectivity of pseudoviruses after depleting by antibody or serum IgG

Pseudovirus	Depleting Antibody	% Loss of infectivity
Wild type	HVTR88	65 ± 10
	S309	70 ± 10
	CR3022	25 ± 10
	COVA2-17	75 ± 10
	HCS79 (serum IgG)	60 ± 20
	HCS84 (serum IgG)	70 ± 20
Alpha	HVTR88	80 ± 10
	S309	80 ± 10
	COVA2-17	70 ± 10
	HCS79 (serum IgG)	90 ± 10
	HCS84 (serum IgG)	80 ± 10
Beta	COVA2-15	50 ± 10
	HVTR88	60 ± 10
	S309	70 ± 10
	HCS84 (serum IgG)	70 ± 20
	HCS79 (serum IgG)	70 ± 5
Delta	COVA 2-15	50 ± 10
	HVTR-88	80 ± 5
	S309	75 ± 10
	HCS84 (serum IgG)	70 ± 10
	HCS79 (serum IgG)	70 ± 10
BA.1	S309	80 ± 20
	CS34.1 (serum IgG)	nd
BA.5	S309	60 ± 20
	CS34.1 (serum IgG)	nd
	CS35.2 (serum IgG)	nd

nd = not done
