# Supplementary material for: Spike conformational and glycan heterogeneity associated with furin cleavage causes incomplete neutralization of SARS-CoV-2
Source: Nat Commun. 2025 Nov 19;16:10130. doi: 10.1038/s41467-025-65099-y (PMC12630640; doi:10.1038/s41467-025-65099-y)
Supplement: Supplementary file 2 — Reporting summary [file 41467_2025_65099_MOESM2_ESM.pdf]

Corresponding author(s): Rajesh P. Ringe
Last updated by author(s): Oct 3, 2025

Reporting Summary

Nature Portfolio wishes to improve the reproducibility of the work that we publish. This form provides structure for consistency and transparency in reporting. For further information on Nature Portfolio policies, see our Editorial Policies and the Editorial Policy Checklist.

Statistics

For all statistical analyses, confirm that the following items are present in the figure legend, table legend, main text, or Methods section.

n/a	Confirmed	
☐	☒	The exact sample size (*n*) for each experimental group/condition, given as a discrete number and unit of measurement
☐	☒	A statement on whether measurements were taken from distinct samples or whether the same sample was measured repeatedly
☐	☒	The statistical test(s) used AND whether they are one- or two-sided<br>*Only common tests should be described solely by name; describe more complex techniques in the Methods section.*
☒	☐	A description of all covariates tested
☒	☐	A description of any assumptions or corrections, such as tests of normality and adjustment for multiple comparisons
☐	☒	A full description of the statistical parameters including central tendency (e.g. means) or other basic estimates (e.g. regression coefficient) AND variation (e.g. standard deviation) or associated estimates of uncertainty (e.g. confidence intervals)
☐	☒	For null hypothesis testing, the test statistic (e.g. *F*, *t*, *r*) with confidence intervals, effect sizes, degrees of freedom and *P* value noted<br>*Give P values as exact values whenever suitable.*
☒	☐	For Bayesian analysis, information on the choice of priors and Markov chain Monte Carlo settings
☒	☐	For hierarchical and complex designs, identification of the appropriate level for tests and full reporting of outcomes
☒	☐	Estimates of effect sizes (e.g. Cohen's *d*, Pearson's *r*), indicating how they were calculated

Our web collection on statistics for biologists contains articles on many of the points above.

Software and code

Policy information about availability of computer code

Data collection	No software was used for data collection.
Data analysis	GraphPad Prism version 8.4.3, ImageJ 1.54g

For manuscripts utilizing custom algorithms or software that are central to the research but not yet described in published literature, software must be made available to editors and reviewers. We strongly encourage code deposition in a community repository (e.g. GitHub). See the Nature Portfolio guidelines for submitting code & software for further information.

Data

Policy information about availability of data

All manuscripts must include a data availability statement. This statement should provide the following information, where applicable:

- Accession codes, unique identifiers, or web links for publicly available datasets
- A description of any restrictions on data availability
- For clinical datasets or third party data, please ensure that the statement adheres to our policy

All data supporting the findings of this study are available within the paper and its Supplementary Information.

Research involving human participants, their data, or biological material

Policy information about studies with human participants or human data. See also policy information about sex, gender (identity/presentation), and sexual orientation and race, ethnicity and racism.

Reporting on sex and gender	No sex- or gender-based anlyses were performed as it not relevant in this study.
Reporting on race, ethnicity, or other socially relevant groupings	This study is not related to race, ethnicity, or other socially relevant groupings.
Population characteristics	See above
Recruitment	The selection of the participants who recovered from COVID-19 were selected to assess SARS-CoV-2 neutralizing activity. The selection of the blood donors were based on confirmed infection by RT-PCR test and the period of infection in the course of pandemic.
Ethics oversight	The human serum samples were collected in this study and the protocol was approved by Institutional Ethics Committee of CSIR-Institute of Microbial Technology (Protocol number - IEC May 2020#2).

Note that full information on the approval of the study protocol must also be provided in the manuscript.

Field-specific reporting

Please select the one below that is the best fit for your research. If you are not sure, read the appropriate sections before making your selection.

☒ Life sciences ☐ Behavioural & social sciences ☐ Ecological, evolutionary & environmental sciences

For a reference copy of the document with all sections, see nature.com/documents/nr-reporting-summary-flat.pdf

Life sciences study design

All studies must disclose on these points even when the disclosure is negative.

Sample size	More than five samples were analysed for statistical significance which was sufficient to estimate statistical differences in neutralization sensitivity if any.
Data exclusions	No data were excluded from analysis
Replication	All experiments were done in technical and biological repeats and good reproducibility was observed.
Randomization	This is not relevant as the study was focused on protein conformational aspects. This study is not a clinical trial.
Blinding	Blinding was not related to this study as this study is not a clinical trial or related to clinical trial in any way.

Reporting for specific materials, systems and methods

We require information from authors about some types of materials, experimental systems and methods used in many studies. Here, indicate whether each material, system or method listed is relevant to your study. If you are not sure if a list item applies to your research, read the appropriate section before selecting a response.

Materials & experimental systems

n/a	Involved in the study	
☐	☒	Antibodies
☐	☒	Eukaryotic cell lines
☒	☐	Palaeontology and archaeology
☒	☐	Animals and other organisms
☒	☐	Clinical data
☒	☐	Dual use research of concern
☒	☐	Plants

Methods

n/a	Involved in the study	
☒	☐	ChIP-seq
☒	☐	Flow cytometry
☒	☐	MRI-based neuroimaging

Antibodies

Antibodies used	COVA2-15, HVTR4, HVTR88, HVTR11, S309, COVA1-16, COVA309-35, ADG20, 4A8, COVA2-17, COVA1-22, CR3022, S1-49 (nanobody),

Antibodies used	anti-nucleocapsid rabbit monoclonal (Abcam, catlog # EPR24334-118), rabbit anti-Spike (Abcam, Catlog # Ab272504), Anti-nucleocapsid (Abcam, catlog # Ab271180)
Validation	All the following non-commercial antibodies were obtained using synthetic DNA constructs based on the original sequence. All the antibodies were expressed in expi293 cells and were confirmed by gel electrophoresis and their binding to the antigen. The commercially obtained antibodies were used according to product data sheet.<br>COVA2-15, COVA1-16, COVA2-17, COVA1-22 were originally described in Brouwer et. al. Science. 2020 Aug 7;369(6504):643-650.<br>COVA309-35 is described in Guerra et. al. iScience. 2023 Sep 22;26(10):108009<br>HVTR4, HVTR88 are described in Hingankar et. al. PLoS Pathog. 2022 Apr 28;18(4):e1010465<br>HVTR11 is described in Deshpande et. al. Microbiol Spectr. 2023 Mar 22;11(2):e0433222<br>S309 is Sotrovimab and originally described in Pinto et. al. Nature. 2020 Jul;583(7815):290-295<br>ADG20 is originally described in Yuan et. al. Proc Natl Acad Sci U S A. 2022 Jul 19;119(29):e2205784119<br>4A8 is described in Chi et. al. Science. 2020 Aug 7;369(6504):650-655<br>CR3022 antibody is described in Yuan et. al. Science. 2020 May 8;368(6491):630-633<br>S1-49 nanobody is originally described in Mast et. al. Elife. 2021 Dec 7;10:e73027

Eukaryotic cell lines

Policy information about cell lines and Sex and Gender in Research

Cell line source(s)	HEK293T-ACE2, HEK293T-ACE2-TMPRSS2 cells were obtained from BEI resources and cat numbers mentioned in the manuscript. Vero E6-TMPRSS2 cells were obtained from JCRB cell bank (JCRB #1818). HEK293T, A549 and VeroE6 cells were obtained from ATCC.
Authentication	The cell lines are authenticated at the original source. None of the cell lines were authenticated at the user location.
Mycoplasma contamination	All cell lines tested negative for mycoplasma contamination.
Commonly misidentified lines (See ICLAC register)	No commonly misidentified cell line was used in this study.

Plants

Seed stocks	No seed stock was used in this study.
Novel plant genotypes	No plant was used in this study.
Authentication	No seed stock or plant was used in this study.
